# Long-term effects of maternal calcium supplementation on childhood growth differ between males and females in a population accustomed to a low calcium intake

**DOI:** 10.1016/j.bone.2017.06.001

**Published:** 2017-10

**Authors:** Kate Anna Ward, Landing Jarjou, Ann Prentice

**Affiliations:** a Nutrition and Bone Health, Medical Research Council Elsie Widdowson Laboratory, Cambridge, UK; b MRC Lifecourse Epidemiology, University of Southampton, Southampton, UK; c Calcium, Vitamin D and Bone Health, MRC Keneba, MRC Unit The Gambia, Gambia

**Keywords:** Calcium, Growth, Child, Maternal, Bone, Body composition

## Abstract

The importance of adequate calcium intakes for healthy growth and bone development has long been recognised. Recent evidence suggests that calcium supplementation may have sex-specific effects on bone growth in childhood. The aim was to describe the long-term effects of calcium supplementation in pregnant Gambian women with a low calcium intake (ISCRTN96502494) on offspring height, weight, bone and body composition in childhood, and whether the effects differ by sex.

Children of mothers who participated in the original calcium supplementation trial were measured at age 8–12 years using dual-energy X-ray absorptiometry and peripheral quantitative computed tomography. Linear models tested for sex*supplement interactions before and after adjusting for current age and size in early life.

447 children, aged 9.2(SD 0.9) years, were measured. Significant sex*supplement interactions (*p* < 0.05) were observed for many of the anthropometric and bone outcomes, Females whose mothers received calcium (F-Ca) were shorter, lighter with smaller bones and less bone mineral than those whose mothers received placebo (F-P), differences (SE) ranged from height = − 1.0 (0.5)% to hip BMC − 5.5 (2.3)%. Males from mothers in the calcium group (M-Ca) had greater mid-upper arm circumference (MUAC) (+ 2.0 (1.0)%, *p* = 0.05) and fat mass (+ 11.6 (5.1)%, *p* = 0.02) and tended towards greater BMC and size than those whose mothers were in the placebo group (M-P). The differences in anthropometry and body composition were robust to adjustment for current height and weight, whereas all bone differences became non-significant. F-P were taller with more BMC than M-P, whereas F-Ca had similar sized bones and mineral content to M-Ca.

Calcium supplementation of pregnant women with low calcium intakes altered the childhood trajectories of growth and bone and body composition development of their offspring in a sex-specific manner, resulting in slower growth among females compared to placebo and accelerated growth among males by age 8–12 years.

## Highlights

• Maternal Ca supplementation affects child growth in rural Gambia.
• Calcium offspring girls grew slower than placebo girls, boys tended to grow faster.
• Size-adjustment attenuated differences in bone outcomes.
• Lean and fat mass differences remained after height correction.
• Prenatal Ca nutrition can modify childhood growth after infancy.

## Graphical abstract

**Figure:**
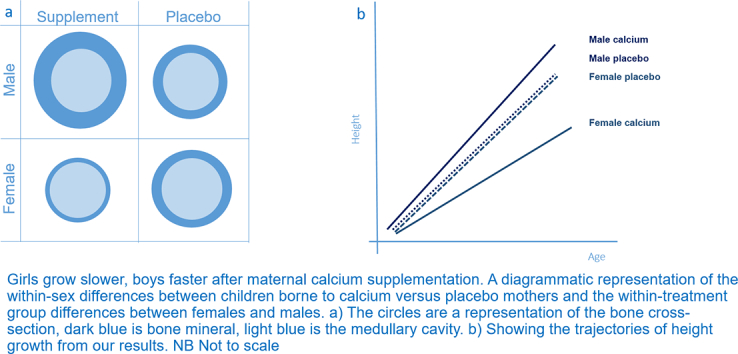

## 1 Introduction

Childhood growth encompasses linear growth (stature), bone accrual (both in width and mineral accumulation) and growth of the tissue compartments and organs (somatic growth). The importance of adequate dietary calcium (Ca) intakes for healthy skeletal growth has long been recognised. Despite this, many trials have not shown significant effects of Ca supplementation on bone growth and mineralisation [1] and those that have shown effects often do not have a sufficient follow-up period to determine whether the effects are sustained. One reason for this may be that the trials are mostly conducted in countries where intakes are, on average, in alignment with dietary recommendations [2]. Less is known from populations where habitual Ca intakes are low. Also, it is not known whether changes in Ca intake at different stages of childhood and adolescence have differential effects on longitudinal and appositional skeletal and somatic growth, and whether the response to intervention differs between males and females [3], [4], [5].

Evidence from our studies in rural Gambia, where dietary Ca intakes are very low, have suggested that Ca supplementation may have unexpected effects depending on the stage of life and in a sex-specific manner. In a Ca supplementation trial (ISRCTN28836000) of pre-pubertal children with low habitual dietary Ca intakes of around 300 mg/day, we showed that the timing of the pubertal growth spurt was brought forward in males who had received a Ca carbonate supplement for 12 months at age 8–12 years, such that their height and bone development were greater in mid-adolescence than males in the placebo group [6], [7], whereas no such effects were observed in the females [6]. Continued follow-up of the males showed that those who had received the calcium supplement pre-puberty stopped growing earlier, and were significantly shorter (3.5 (SE 1.1) cm) at the end of growth than those who had been in the placebo group [6]. In addition, the short-term increases in bone mineral content (BMC) and bone area (BA) in these males due to Ca supplementation were attenuated [6], [7], [8], [9].

In a second study, a trial of maternal Ca supplementation during pregnancy (ISCRTN96502494), we reported that, contrary to expectations, mothers who received a daily Ca carbonate supplement from 20 weeks gestation to term mobilised more bone mineral during lactation than those who received placebo [10], [11], resulting in lower BMD that was still evident 5 years post supplementation [11]. There were no supplement effects observed on the size of their offspring at birth or during 12 months post-partum [12], [13]. There were also no effects seen on infant whole body and radial BMC measured in a sub-set, although a weakly significant group effect was observed whereby the whole body BMC and BA of the infants of mothers who had received Ca supplement had increased more slowly by 12 months than those of the infants of mothers in the placebo group [10], [12], [13]. Males were significantly heavier at 2 weeks and longer at 52 weeks than females. At age 8–12 years, there were no significant differences between males and females in the cohort in height, weight or body mass index. Girls had greater fat mass, whole body and spine BMC than the boys but lower lean mass, bone area and BMC at the hip [14]. Because males were heavier at 2 weeks and longer at 52 weeks, and that there were no differences in height and weight at age 8–12 years, the data demonstrate different rates of growth in males and females.

The aim of the current study was to determine whether there were lasting effects of the maternal Ca supplementation in this trial on the growth, bone development and body composition of the offspring when the children were aged 8–12 years, prior to the adolescent growth spurt. This was assessed using anthropometry, dual-energy X-ray absorptiometry (DXA) and peripheral quantitative computed tomography (pQCT). The hypothesis was that the children of mothers who received Ca supplementation would be taller and have higher BMC and bone mineral density (BMD) than those whose mothers had been in the placebo group, and that these differences would differ by sex. Our secondary aims were to determine the effects of maternal Ca supplementation on the tibial cortical and trabecular bone compartments and on body composition (fat and lean masses, mid-upper arm circumference and triceps skinfold thickness).

## 2 Subjects and methods

### 2.1 Subjects

This study was conducted at MRC Keneba, West Kiang, The Gambia. All children, whose mothers had taken part in the supplementation trial of Ca in pregnancy (ISCRTN96502494) and had delivered a healthy baby, were invited to participate when aged 7.8 to 11.9 years. Details of the trial have been previously published [12], [13]. Briefly, recruitment was in three ante-natal clinics serving 16 villages. Randomisation was stratified by antenatal clinic in blocks of four to minimise bias and potential confounding by season. Pregnant mothers in the supplement group received 1500 mg Ca as Ca carbonate (3 tablets of Calcichew ™, Calcichew; Nycomed Pharma AS, Asker, Norway; distributed in the United Kingdom by Shire Pharmaceutical Development Ltd., Andover, UK), or a matching placebo, daily from 20 weeks of pregnancy until term. Mean (SD) maternal dietary Ca intakes during the trial were 1831 (177) mg/day in the calcium group and 356 (159) mg/day in the placebo group [13].

Measurement visits were scheduled to ensure equal distribution of children across the age-range and study period, and took place during the dry season, a time of year when food shortages, malaria and infectious illnesses are less prevalent. The study was approved by the Joint MRC/Gambian Government Ethics Committee and informed written consent was obtained from the parent or guardian of each child.

### 2.2 Anthropometry

Standing height was measured to the nearest 0.1 cm using a stadiometer (SECA 225, Birmingham, UK). Height-for-age z-scores (HAZ) as an indicator of maturity were calculated using WHO growth references [15]. Weight was measured on electronic scales (Tanita HD310, Amsterdam, The Netherlands) to the nearest 0.1 kg, with the subject wearing light clothing and no shoes. Mid-upper arm circumference (MUAC) and triceps skinfold thickness (TST) were measured at the mid-point of the upper left arm using a non-stretchable tape measure and a skinfold calliper (Holtain Ltd., UK) respectively. Data were available from the original trial on length, weight and head circumference at birth and during infancy [12].

### 2.3 Dual energy X-ray absorptiometry (DXA)

Bone and body composition measurements were obtained using a GE Lunar Prodigy DXA scanner, software version 10.51.006 (GE Medical Systems, GE Lunar Corporation, Madison, USA). Outcome measures were whole body less head (WB) [16], lumbar spine (LS), total hip BMC (g) and BA (cm^2^). Lean and fat mass (g) measurements were obtained from the whole body scan. At MRC Keneba, the precision of repeated measurements of aBMD at different skeletal sites in 35 adults, measured twice with repositioning, was: whole body 0.6%, lumbar spine 0.8% and total hip 0.7%.

### 2.4 Peripheral quantitative computed tomography (pQCT)

A Stratec XCT-2000 scanner (Stratec Medizintechnik, Pforzheim, Germany), was used to obtain measurements of the metaphyseal (8%) and diaphyseal (50%) tibia. Measurements were taken using a voxel size 0.5 mm, slice thickness 2 mm and scan location was determined by placing the reference line on the distal border of the tibia endplate. Outcome measures were at the 8% site; total volumetric BMD (mg/cm^3^) and total cross-sectional area (mm^2^), and at the 50% site; tibia cross-sectional area (mm^2^), cortical BMC (mg/mm) and cortical area (mm^2^). Metaphyseal scans were analysed using CALCBD, contour mode 1, peel mode 1, threshold 180 mg/cm^3^, and at the diaphysis, separation mode 1, threshold 710 for cortical content and area and 280 mg/cm^3^ for total area. The precision of repeated measures in adults (*n* = 35, measured twice with repositioning) at our centre was < 1% for all outcomes.

### 2.5 Statistical analysis

Statistical analysis was performed using the Linear Model facility in Data Desk 6.1.1 (Data Description, Ithaca, NY). Summary data are presented as mean (SD) or median (interquartile range). Sex differences in the early life variables of participants in the current study were tested for using one-way ANOVA except for maternal parity at the time of the trial and season of birth of the offspring, where the Chi-square test was used. There was no evidence of a supplement effect or a sex*supplement interaction in any of the early life variables.

Sex-specific supplement effects on each outcome variable were tested for using multiple regression and analysis of covariance, by including a sex*supplement interaction term in all models. Variables were converted to natural logarithms prior to statistical modelling, whereby, for discrete variables, difference × 100 corresponds closely to percentage difference [(difference/mean) × 100] [17]. Scheffé post-hoc tests were used to adjust for multiple testing and to report differences between sex*supplement groups. The four sex*supplement groups were: males whose mothers had been in the calcium supplement group in pregnancy (M-Ca); females of mothers in the calcium group (F-Ca); males whose mothers had been in the placebo group in pregnancy (M-P) and females of mothers in the placebo group (F-P). Summary data on differences between males (M) and females (F), between maternal supplement groups (Ca/P) and between the four sex*supplement groups are presented as mean percentage difference (SE).

To consider the best approach to account for possible confounding on childhood growth by inter-individual variation in size in early life, independent of maternal Ca supplementation, a preliminary series of separate models was developed that included one of the following measurements: weight, length, mid-upper arm circumference and head circumference at birth, 2 weeks and 12 months postpartum. Each measure had a similar effect on reducing the residual variance of the models, but length at 52 weeks gave the greatest reduction. For this reason length at 52 weeks was used in all subsequent models as the surrogate to adjust for inter-individual differences in size at birth and during infancy. The sex and supplement differences in outcome variables at 8–12 years of age can therefore be considered to represent differences in growth since infancy. Models without length at 52 weeks (Table 2) gave similar results for sex*supplement interactions, albeit with attenuated significance because of the greater residual variance, and the magnitude of differences between supplement groups within each sex was also similar. The magnitude of the observed sex differences within each supplement group were, however, generally smaller without length at 52 weeks in the models, because the males were bigger than the females in early life [12] but not at age 8–12 years [14].

Two models were constructed to test for sex*supplement effects on the growth of the children at 8–12 years. The first model included length at 52 weeks, current age, sex (M/F), maternal supplement group (Ca/P) and a sex*supplement group interaction. The second model was based on the first but adjusted the bone and body composition data for current body size, using height and weight for bone variables, and height for lean and fat masses. DXA-measured BMC was adjusted for BA in addition to weight and height (size-adjusted BMC, SA-BMC) [18]. To consider possible effects of birth order and season of birth on child growth, maternal parity at the time of the trial (parous/nulliparous) and season of birth (wet (July-Dec) or dry (Jan-Jun)) were added as dichotomous variables [12]. Neither birth order, parity nor season of birth appreciably changed the results or conclusions drawn and are not included in the analysis presented in this paper.

The number of individuals available for inclusion for the current study was pre-determined by the sample size of the maternal supplementation trial. 100 children per group were sufficient to detect a difference between pairs of groups of 0.4 SD in each outcome variable, with 80% power and an alpha of 0.05.

## 3 Results

**Fig. 1:**
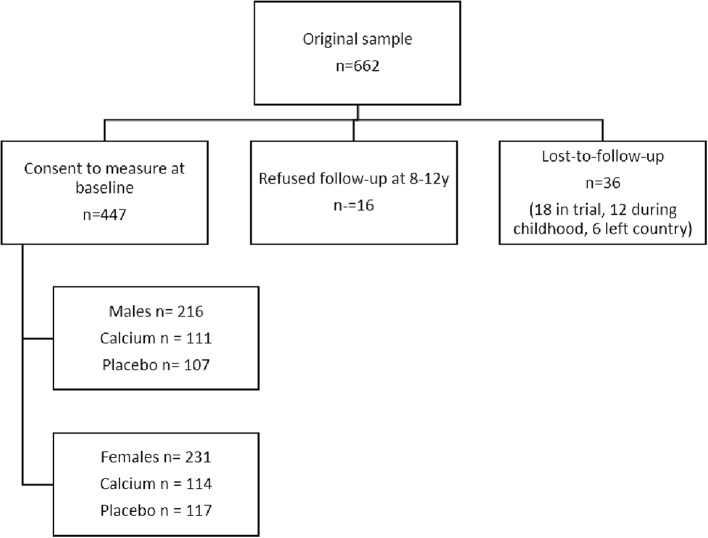
CONSORT diagram showing reasons for loss to follow-up from the trial of maternal calcium supplementation during pregnancy. Of the 662 women who were recruited, the offspring of 447 were recruited to this study.

Four hundred and forty-seven children were recruited to the study and attended for measurement at the clinic; 216 M and 231 F, mean (SD) age 9.3 (0.1) years and 9.2 (0.1) years respectively. Fig. 1 illustrates the derivation of the sample from the maternal cohort. Descriptive statistics for the general characteristics of the study population, by sex and by maternal supplement group, are given in Table 1. Males were heavier at 2 weeks of age and longer at 52 weeks of age than females. Maternal parity at the time of the trial did not differ between males and females nor did season of birth. Table 2 gives the descriptive statistics of the bone outcomes and reports the sex*supplement interactions and within-treatment group sex differences. Table 3 presents the main results of the sex*supplement interaction showing mean differences between the supplement and placebo groups by sex.

**Table 1:** Participant age and early life characteristics by sex and supplement group.

	Female		Male		Female vs male difference a
Outcome	Calcium (*n* = 114)	Placebo (*n* = 117)	Calcium (*n* = 109)	Placebo (*n* = 107)	*p* =
Age (y)	9.21 (0.91)	9.20 (0.87)	9.22 (0.90)	9.25 (0.87)	0.7
Weight at 2 weeks (kg)	2.86 (0.34)	2.80 (0.32)	2.91 (0.38)	3.03 (0.39)	*p* < 0.0001
Length at 2 wk. (cm)	48.4 (21)	48.3 (21)	48.7 (28)	49.1 (26)	
Length at 52 wk. (cm)	70.5 (27)	70.6 (31)	72.2 (30)	72.7 (35)	*p* < 0.0001
Parity^b^	4 (2–6)	4 (2–6)	3 (2–6)	4 (2–6)	0.7
Season of birthb,^c^					
*Wet*	61	56	67	60	0.7
*Dry*	69	81	63	69	

Data are mean (SD) except parity which is median (inter-quartile range).

a Differences tested between male and females using one-way ANOVA with continuous variables transformed to natural logarithms. There were no significant sex*supplement interactions in the early life variables.

b Differences tested using Chi-square tests. For maternal parity the variable was dichotomised as nulliparous versus parous at the time of the supplementation trial.

c Wet season was defined as July to December and the dry season, January to June.

**Table 2:** Descriptive characteristics of the cohort by supplement group and sex, superscripts indicate sex differences by supplement group, before and after adjustment for size in early life.

		Calcium		Placebo		Sex difference p a	
	Outcome	Female	Male	Female	Male	F-Ca vs M-Ca	F-P vs M-P
Anthrops							
	Height (m)	1.28 (0.66)	1.28 (0.65)	1.30 (0.71)	1.27 (0.63)	c	d
	Height-age-z-score	− 1.09 (0.79)	− 1.03 (0.85)	− 0.83 (0.91)	− 1.08 (0.78)	c	d,^e^
	Weight (kg)	23.6 (3.5)	24.0 (3.7)	24.4 (4.4)	23.6 (3.3)		d,^e^
	MUAC (mm)	170 (14)	168 (14)	173 (17)	166 (12)	c	d,^e^
	Triceps Skinfolds	74.7 (17.6)	61.8 (14.4)	76.6 (22.3)	58.8 (11.9)	b,^c^	d,^e^
DXA	*Whole body*						
	BMC f (kg)	5.79 (1.33)	6.13 (1.36)	6.02 (1.37)	6.00 (1.24)	b	e
	Bone area f (m^2^)	8.33 (1.37)	8.65 (1.33)	8.55 (1.36)	8.57 (1.24)	b	e
	Fat mass (kg)	3.40 (1.31)	2.31 (1.04)	3.74 (2.07)	2.04 (0.78)	b,^c^	d,^e^
	Lean mass (kg)	19.1 (2.6)	20.5 (3.0)	19.4 (2.8)	20.5 (2.7)	b,^c^	d
	*Lumbar spine*						
	BMC (g)	16.9 (3.3)	17.3 (3.4)	17.4 (2.9)	16.7 (3.1)		d,^e^
	Bone area (cm^2^)	26.9 (3.1)	28.0 (3.3)	27.2 (2.8)	27.7 (3.1)	b,^c^	
	*Total hip*						
	BMC (g)	10.8 (2.6)	12.1 (2.7)	11.3 (2.4)	12.2 (2.6)	b,^c^	d
	Bone area (cm^2^)	14.9 (2.5)	14.8 (2.2)	15.3 (2.6)	14.9 (2.2)	c	e
pQCT	*Tibia*^g^						
	Tot vBMD (mg/cm^3^)	318 (34)	334 (34)	321 (36)	329 (29)	b,^c^	d,^e^
	Total area (mm^2^)	277 (45)	292 (51)	285 (48)	287 (45)	b	e
	Cortical area (mm^2^)	143.9 (22.3)	155.6 (24.4)	149.9 (24.7)	152.6 (22.5)	b,^c^	e
	Cortical BMC (mg/mm)	155.1 (25.6)	166.6 (27.3)	161.0 (28.4)	163.5 (25.7)	b,^c^	e

Data are presented as mean (SD).

All continuous variables were transformed to natural logarithms for the models.

Number of missing measurements: DXA: < 3 per group, per site; pQCT < 12 per group 8% tibia, < 5 per group 50% tibia.

a Significance of sex differences within treatment group at *p* < 0.05 are reported from Scheffe's post hoc tests in 1) a linear model including sex, supplement group, current age and sex*supplement interaction and 2) as 1) and also including length at 52 weeks. All continuous variables were transformed to natural logarithms for the models. Number of missing measurements: DXA: < 3 per group, per site; pQCT < 12 per group 8% tibia, < 5 per group 50% tibia. Significant sex differences within treatment group at *p* < 0.05 are reported from Scheffe's post hoc tests in Model 1, a linear model including sex, supplement group, current age and sex*supplement interaction, and in Model 2, a linear model including the variables in Model 1 plus length at 52 weeks.

b Female calcium (F-Ca) vs. male calcium (M-Ca), Model 1;

c Female calcium (F-Ca) vs. male calcium (M-Ca), Model 2;

d Female placebo (F-P) vs. male placebo (M-P), Model 1;

e Female placebo (F-P) vs. male placebo (M-P), Model 2.

f Whole body less head measurements were used for BMC and BA.

g Tibia measurements, total vBMD, total volumetric bone mineral density at the distal tibia (8% site); total area, cortical area and BMC are measured at the 50% tibia diaphysis.

**Table 3:** Mean (SE) percent differences between the supplement and placebo groups, split by sex^a^.

	Model 1^b^					Model 2^c^				
	Females		Males		Sex*supplement interaction *p* =	Females		Males		Sex*supplement interaction *p* =
	Mean difference (SE) %	*p*-Value	Mean difference (SE) %	*p*-Value		Mean difference (SE) %	*p*-Value	Mean difference (SE) %	*p*-Value	
Height	− 1.0 (0.5)	*0.04*	0.5 (0.5)	*0.3*	***0.03***	–	–	–	–	–
HAZ^d^	− 0.2 (0.1)	*0.04*	0.1 (0.1)	*0.3*	***0.02***	–	–	–	–	–
Weight	− 3.3 (1.5)	*0.03*	2.2 (1.6)	*0.2*	***0.01***	–	–	–	–	–
MUAC^e^	− 1.8 (1.0)	*0.04*	2.0 (1.0)	*0.05*	***0.007***	− 0.2 (0.5)	*0.7*	1.0 (0.6)	0.09	0.1
TST^f^	− 1.5 (3.0)	*0.6*	4.7 (3.1)	*0.1*	***0.1***	1.1 (2.5)	*0.7*	3.0 (2.6)	*0.2*	*0.6*
Whole body										
BMC	-4.6 (2.2)	*0.03*	2.8 (2.2)	*0.2*	***0.02***	− 0.3 (0.6)	*1.0*	0.5 (0.6)	*0.4*	*0.5*
Bone area	− 3.3 (1.4)	*0.02*	1.6 (1.5)	*0.3*	***0.02***	− 0.3 (0.7)	*0.6*	− 0.9 (0.8)	*0.9*	*0.8*
Lean mass	− 2.4 (1.4)	*0.08*	1.1 (1.4)	*0.4*	***0.07***	0.4 (1.0)	*0.7*	0.1 (1.0)	*0.9*	*0.7*
Fat mass	− 4.8(4.9)	*0.3*	11.6 (5.5)	*0.02*	***0.02***	− 2.1 (4.8)	*0.7*	10.0 (4.9)	*0.04*	*0.08*
Lumbar spine										
BMC	-4.4 (2.1)	*0.03*	3.9 (2.3)	*0.09*	***0.007***	− 1.5 (0.2)	*0.4*	2.0 (1.8)	*0.3*	*0.2*
Bone area	− 1.9 (1.2)	*0.1*	2.1 (1.3)	*0.1*	***0.03***	− 0.4 (1.0)	*0.7*	1.2 (1.1)	*0.3*	*0.3*
Total hip										
BMC	-5.5 (2.3)	*0.02*	0.5 (2.5)	*0.8*	*0.07*	− 1.6 (1.6)	*0.3*	− 1.7 (1.7)	*0.3*	*0.9*
Bone area	− 3.1 (1.5)	*0.04*	− 0.0 (1.5)	*0.9*	*0.1*	− 0.9 (1.0)	*0.4*	− 1.1 (1.0)	*0.3*	*0.9*
Tibia										
Total vBMD	− 2.8 (4.7)	*0.6*	2.7 (4.7)	*0.6*	*0.4*	− 0.4 (1.5)	*0.8*	0.4 (1.5)	*0.8*	*0.7*
Midshaft CSA	− 3.7 (1.7)	*0.03*	2.2 (1.8)	*0.2*	***0.02***	− 1.2 (1.4)	*0.4*	0.9 (1.4)	*0.5*	*0.3*
BMC	− 3.6 (1.9)	*0.06*	1.7 (1.9)	*0.4*	***0.04***	− 1.2 (1.6)	*0.5*	0.7 (1.7)	*0.7*	*0.4*
Cortical area	− 4.1 (1.7)	*0.02*	1.9 (1.8)	*0.3*	***0.01***	− 1.7 (1.5)	*0.2*	0.8 (1.5)	*0.6*	*0.2*

a Differences between children whose mothers took calcium versus those who had placebo were tested using linear models with the following covariates.

b Sex, supplement, current age, length at 52 weeks, sex*supplement interaction.

c Sex, supplement, current age, length at 52 weeks, sex*supplement interaction, current height and weight, and DXA BMC bone area. All continuous variables were in natural logarithms.

d HAZ height-for-age Z score calculated from WHO reference [15].

e MUAC Mid-upper arm circumference.

f TST Triceps skinfold thickness.

### 3.1 Sex differences within treatment group

Prior to adjustment for size in early life (Table 2), there were no significant sex differences in attained height, HAZ, weight and MUAC at 8–12 years in the Ca group but females were significantly bigger than males in the placebo group. F-Ca tended to have smaller bones with less bone mineral than males in the Ca group, but there were fewer and less consistent sex differences in the placebo group. Females had greater TST and fat mass and less lean mass than males in both supplement groups.

**Fig. 2:**
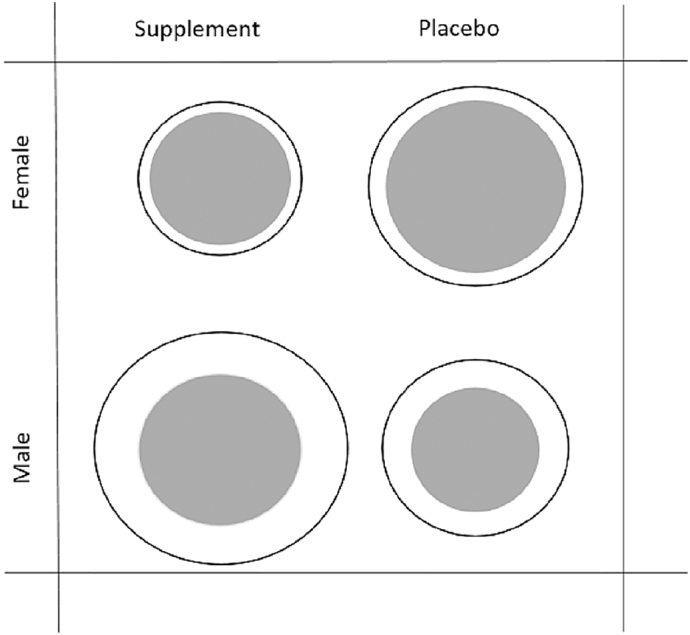
A diagrammatic representation of the within-sex differences between children borne to calcium versus placebo mothers and the within-treatment group differences between females and males. The circles are a representation of the bone cross-section, white is bone mineral, dark grey is the medullary cavity.

After adjustment for length at 52 weeks, females were bigger for their size in early life than males by age 8–12 years in anthropometry and fat mass (F-Ca versus M-Ca: height + 1.1 (0.5)% *p* = 0.02, weight + 1.5 (1.6)% *p* = 0.3, MUAC + 2.1 (1.0)% *p* = 0.04, TST = + 29.9 (3.1)% *p* ≤ 0.001; HAZ + 0.22 (0.10) *p* = 0.02; F-P versus M-P: height + 2.6 (0.5)% *p* ≤ 0.001, weight + 3.3 (1.5)% *p* ≤ 0.001, MUAC + 5.9 (1.0)% *p* ≤ 0.001, TST + 26.1 (3.2)% *p* ≤ 0.001); HAZ + 0.54SD (0.10) *p* ≤ 0.001). The sex differences were similar for most of the bone variables, but less consistently in the Ca than in placebo group (Table 2). Overall the pattern in the bone variables was that F-Ca had smaller bones containing less bone mineral than M-Ca and F-P had larger bones containing less mineral than M-P; depicted in Fig. 2.

### 3.2 Sex-supplement interactions: Adjusted for length at 52 weeks, current age (Table 2, Table 3)

Among the anthropometric variables, after adjustment for current age and size in early life, there were significant sex*supplement interactions for height (*p* = 0.03), HAZ (*p* = 0.02), weight (*p* = 0.01) and MUAC (*p* = 0.007). F-Ca were significantly shorter and lighter than F-P, with a difference in MUAC and TST in the same direction, although not significant. Differences between the supplement groups in M were in the opposite direction to those in F; the pattern was consistent but the difference was only significant for MUAC.

Among the bone variables, there were significant sex*supplement interactions at the whole body, lumbar spine, and tibia (all *p* < 0.05). F-Ca had significantly smaller (− 2 to − 4%) bones containing less bone mineral (− 3 to − 6%) than F-P. At the hip these differences were not significant (BMC, *p* = 0.07) but followed a similar pattern and were of similar magnitude. In contrast, although none were statistically significant, M-Ca tended to have larger bones (+ 1 to + 2%) with greater BMC (+ 1 to + 4%) than M-P. Despite being non-significant, it is noteworthy that the differences between the supplement groups in M were in the opposite direction to those in F and in the same direction as those in the anthropometric variables. There were no significant differences in total or trabecular volumetric BMD at the distal tibia (trabecular vBMD data not shown), a predominantly trabecular site. In contrast, at the diaphysis of the tibia, a cortical site, F-Ca had smaller bones with lower BMC than F-P, consistent with an effect on growth rather than on bone mineralisation.

For the DXA-measured body composition outcomes, there was a significant sex*supplement effect for fat mass (*p* = 0.02), where F-Ca tended towards less fat mass than F-P (*p* = 0.3) whereas M-Ca had significantly greater fat mass than M-P (*p* = 0.02). The sex*supplement interaction for lean mass was of borderline significance (*p* = 0.07) and the pattern of differences between supplement groups within each sex was similar to the bone variables.

### 3.3 Sex-supplement interactions: Size-adjusted model, adjusting for length at 52 weeks, current age, height, weight (bone outcomes only) and BA (DXA-measured BMC only)

In the size-adjusted models all significant sex*supplement interactions for bone outcomes became non-significant and differences between supplement groups in the females were attenuated. Similarly, those for MUAC and body composition were also attenuated (*p* value for sex*supplement interaction: MUAC = 0.04, fat mass *p* = 0.08, lean mass *p* = 0.7). There was a trend to greater MUAC in M-Ca than M-P after height adjustment and a significantly greater fat mass but there were no significant differences in fat mass between F-Ca and F-P. There were no significant differences in height-adjusted lean mass between the supplement groups in either sex.

## 4 Discussion

Our study describes the long-term effects of calcium carbonate supplementation of pregnant mothers on a low calcium diet on the skeletal and somatic growth of their offspring at age 8–12 years. We found significant sex-specific effects on the growth of the children after infancy. Females whose mothers had received the Ca supplement had narrower bones, containing less bone mineral, than the female offspring of mothers who were in the placebo group. They were also significantly shorter, lighter and had less fat mass, but greater lean mass, than females born to mothers in the placebo group. After correction for current body size, the effects were attenuated for the bone variables, lean mass and fat mass, indicating that their skeletal size, bone mineral content and body composition were appropriate for their attained size. Conversely in males, height, weight, MUAC and bone outcomes tended to be greater in those born to mothers in the calcium group, although few of these differences were significant and were attenuated after size correction. In addition, males whose mothers had received the Ca supplement had significantly higher fat mass than those whose mothers were in the placebo group, whereas the groups had similar lean mass. In contrast to the bone and lean mass data, height correction of fat mass measurements in males attenuated only slightly the magnitude of the supplement difference, although the sex*supplement effect was no longer significant.

These results indicate that the maternal calcium supplementation had altered the trajectory of growth differently in females and males such that it resulted in smaller size and fat mass in females and a tendency to greater size and fat mass in males at age 8–12 years. The effects were such that the greater rate of growth of females relative to males at this stage of life, as seen in the placebo group, was diminished in the children of the mothers supplemented with calcium. Rural Gambian children experience maturational delay compared to Western children, and the timing of the pubertal growth spurt is considerably later [6]. Although not assessed directly in this study, the likelihood is that the majority of children were pre-pubertal. However, because females enter puberty earlier than males, the results of our study could suggest that the lasting effect of the the maternal supplement will be sex-specific changes in the timing of puberty, advancing the initiation of the growth spurt in the males and delaying it in the females. Whilst this can only be confirmed by further follow-up the HAZ scores are indicative of this, because as Gambian children become more mature their growth relative to international reference data tends to improve, i.e. exhibit ‘catch-up’ growth [19]. In this study, the difference in HAZ scores between females and males in the calcium group is smaller (0.22 (SD 0.1)) than the placebo group (0.55 (SD 0.1)), indicating less difference in maturity in the calcium groups, likely due to the slower growth in F-Ca, than in the placebo groups.

Our findings of sex differences in the response to maternal Ca supplementation on childhood growth have similarities to those seen in our earlier longitudinal follow-up of a supplementation trial in The Gambia [6], [7]. Pre-pubertal males aged 8–12 years who received a Ca supplement for a year and who were followed to the end of growth were shown to have gone into their pubertal growth spurt earlier and so reached peak velocities for height and bone development at an earlier age than those who were in the placebo group [6], [7]. The Ca group finished growing earlier and were consequently shorter at the end of growth, than those in the placebo group. Growth in weight and lean mass were not significantly affected by the pre-pubertal Ca supplementation. In contrast, we found no significant effect of Ca supplementation in 8–12 year old females on the timing of the pubertal growth spurt, final height or bone outcomes. We considered that this may have been because the early stages of puberty, before physical signs become evident, were already initiated in these females prior to the start of the supplementation [6], [20]. The long-term consequences of these findings cannot be confirmed without further follow-up of the cohort. In the COHORTS consortium a 1SD difference in conditional height growth in mid-childhood had consequences for final height, BMI and blood pressure in young adulthood [21]. Our findings are modest, yet significant, and if they track through to the end of growth may have a significant impact on future health.

Concentrations of umbilical cord IGF-1 and leptin have been linked to offspring growth and bone development in childhood [22], [23]. Both of these factors may be altered by changes in maternal nutritional status and could explain the differences in growth we observed in our study. Most relevant are the findings from a trial in Burkina Faso of maternal supplementation with the multi-micronutrient UNIMMAP (which contains no calcium), where sex differences in concentrations of leptin and IGF-1 at birth were reported [4]. Male offspring of supplemented mothers had higher cord blood leptin and IGF-1 levels than those whose mothers had been in the standard iron-folate group. In females, there were no differences in cord blood IGF-1 concentrations, but leptin was lower. During growth IGF-1 and leptin are markers of pubertal development [24], [25] and if such a pattern of hormonal changes due to maternal supplementation were to continue in post-natal life, it is possible that it would be associated with accelerated growth and maturation in males, and conversely, would be associated with delayed growth and maturation in females, consistent with our findings. The Burkino Faso study did not report whether the observed biochemical effects of the maternal micronutrient supplementation differed by sex of the infant. However, the sex differences we report in growth and bone development in response to maternal Ca supplementation are consistent with previous studies that have suggested that female and male offspring differ in their susceptibility to changes in maternal diet [3], [4], [5], [26], possibly through differences in epigenetic modifications [27].

The primary aim of this work was to determine the effects of maternal Ca supplementation on offspring growth and bone development and to consider whether there were differences by sex. To our knowledge, our study is the first to consider whether maternal micronutrient supplementation has long-term effects on childhood growth and bone development using gold-standard methods of bone and body composition measurements in addition to anthropometry. In addition, few trials have tested for a sex difference in offspring growth and bone development following maternal supplementation. Previous studies in populations or groups with low calcium intakes have linked maternal Ca supplementation, or habitual Ca intake, to offspring growth and/or BMC in infancy and early childhood [28], [29], although the findings are inconsistent and sex differences in supplement response were not considered. In a Ca supplementation trial in the US, infant BMC was greater in mothers with a baseline calcium intakes < 600 mg/day who received Ca supplements than in the placebo group and this difference was robust to adjustment for child size [28]. A study from India, where habitual Ca intakes are similar to those in The Gambia, maternal Ca intake was positively related to infant BMC [29]. In contrast, in our trial of maternal Ca supplementation of Gambian mothers, we found no evidence of an effect on fetal or infant growth, with the possible exception of a slower rate of whole body bone mineral accrual by 12 months [12], [13], [30].

Trials of maternal supplementation with micronutrients other than Ca have also shown inconsistent effects on long-term offspring growth and bone development. For example, multiple micronutrient supplementations of Nepalese mothers resulted in an increased birthweight. Neither length nor head circumference were affected in infancy but at 2–3 years of age the children of supplemented mothers were heavier with greater circumferences of the head, chest and mid-upper arm and triceps skinfold thickness than those of the control group [31], [32]. In the UNIMMAP trial in Burkino Faso, the offspring of supplemented mothers had greater height, weight-for-length and head circumference during infancy than those of mothers in the control group, but these differences had mostly disappeared by 30 months of age [32]. A meta-analysis of all UNIMMAP trials with long-term follow-up, however, did not find an overall effect of supplementation on several health outcomes, including anthropometry or body composition [33], [34].

The main limitation of our study is that we do not have pubertal assessments or biochemical data to confirm our findings or hypothesis regarding pubertal timing. Also, the study is a post-hoc study of a trial that had been designed to detect differences in maternal pre-eclampsia rate and infant growth in response to Ca supplementation and was not designed to test sex*supplement differences.

In conclusion, our findings show that early-life events, specifically maternal Ca supplementation, in a rural African community where habitual daily Ca intakes are extremely low, affected childhood growth and bone development in a sex-specific manner at age 8–12 years, an effect that was consistent across several independent measures. Further study is required to investigate mechanisms and consider long-term consequences on health.

**Abbreviations**
BMD: bone mineral density
BMC: bone mineral content
Ca: calcium
BA: bone area
XSA: cross sectional area
DXA: dual energy X-ray absorptiometry
pQCT: peripheral quantitative computed tomography
F-Ca: females whose mothers received calcium supplementation
F-P: females whose mothers received placebo tablets
M-Ca: males whose mothers received calcium supplementation
M-P: males whose mothers received placebo tablets
HAZ: height-for-age *Z*-score
MUAC: mid-upper arm circumference
TST: triceps skinfold thickness

## Funding

This research is jointly funded by the Medical Research Council (MRC) and the Department for International Development (DFID) under the MRC/DFID Concordat agreement programme numbers U105960371; U123261351; MC-A760-5QX00.
